# Close of Volume Fifteen

**Published:** 1876-07

**Authors:** 


### Editorial.

With our next number we commence the sixteenth volume of the new series of THE BUFFALO MEDICAL AND SURGICAL JOURNAL. The JOURNAL has now passed through thirty years of publication since its first issue in 1845, by Prof. Austin Flint, M. D. During that time it has given to its readers some of the most important and valuable facts which have been added to professional knowledge. It was our intention to lay before our readers a brief resumé of some of the more important contributions to medical science which have enriched its pages, and have become in this way the property of the whole world, but we find the task too extensive, we can only mention a few. In these pages Dr. Flint published the first series of cases demonstrating that Typhoid Fever was diffused by impure drinking-water, here were commenced those articles upon the treatment of Fractures and Dislocations by Dr. Hamilton, which have since made his name famous and have grown into a Treatise which is classical. Dalton and Flint, Jr., made in the BUFFALO MEDICAL JOURNAL, a commencement to those contributions to Physiological science which they have since so gloriously continued. The fact that the uterus could be successfully restored to its normal position after years of inversion was here triumphantly demonstrated by Prof. White in a series of cases which have no rival the world over. The method of removing Ovarian Tumors by Enucleation was first explained through our pages, and has since become a recognized operation by all gynæcological surgeons. Time and space will not permit us to indulge in a retrospect such as we would desire, nor is it necessary, the work of the JOURNAL is before its readers, and will speak for itself. We will be pardoned however, if we quote the words of Dr. Sanford B. Hunt, Editor of the *Newark Daily Advertiser*, in a recent address. "The noblest, most patient, most lasting and effective books in American Literature, appeared as serials in THE BUFFALO MEDICAL JOURNAL, or were filled up and rounded afterwards, from that source."

In the coming volume we hope to place before our readers a series of original communications, which shall equal any which have been published since the commencement of the Journal. Care and discretion will also be exercised in the selection of material for the miscellaneous department, to present that only which shall be of practical value. Our large number of readers, widely scattered as they are, must daily observe cases which are of interest and value, we invite them to freely make use of our pages to communicate their ideas. It is by the mental interchange of thought in this manner that knowledge in our profession is destined to grow, and every man should remember the debt he owes to his profession and make frequent payments. B.

